# Microwave Bone Imaging: A Preliminary Investigation on Numerical Bone Phantoms for Bone Health Monitoring

**DOI:** 10.3390/s20216320

**Published:** 2020-11-05

**Authors:** Bilal Amin, Atif Shahzad, Martin O’Halloran, Muhammad Adnan Elahi

**Affiliations:** 1 Electrical and Electronic Engineering, National University of Ireland Galway, H91 TK33 Galway, Ireland; martin.ohalloran@nuigalway.ie (M.O.); adnan.elahi@nuigalway.ie (M.A.E.); 2 Translational Medical Device Lab, National University of Ireland Galway, H91 TK33 Galway, Ireland; 3 School of Medicine, National University of Ireland Galway, H91 TK33 Galway, Ireland; atif.shahzad@nuigalway.ie

**Keywords:** bone health, bone phantoms, compressed sensing, dielectric properties, distorted Born iterative method, microwave tomography

## Abstract

Microwave tomography (MWT) can be used as an alternative modality for monitoring human bone health. Studies have found a significant dielectric contrast between healthy and diseased human trabecular bones. A set of diverse bone phantoms were developed based on single-pole Debye parameters of osteoporotic and osteoarthritis human trabecular bones. The bone phantoms were designed as a two-layered circular structure, where the outer layer mimics the dielectric properties of the cortical bone and the inner layer mimics the dielectric properties of the trabecular bone. The electromagnetic (EM) inverse scattering problem was solved using a distorted Born iterative method (DBIM). A compressed sensing-based linear inversion approach referred to as iterative method with adaptive thresholding for compressed sensing (IMATCS) has been employed for solving the underdetermined set of linear equations at each DBIM iteration. To overcome the challenges posed by the ill-posedness of the EM inverse scattering problem, the$\;L_{2}$-based regularization approach was adopted in the amalgamation of the IMATCS approach. The simulation results showed that osteoporotic and osteoarthritis bones can be differentiated based on the reconstructed dielectric properties even for low values of the signal-to-noise ratio. These results show that the adopted approach can be used to monitor bone health based on the reconstructed dielectric properties.

## 1. Introduction

Microwave imaging (MWI) is an emerging diagnostic technology being investigated for a range of medical applications. The key advantages of MWI for diagnosing and monitoring various diseases compared to existing imaging modalities are non-ionizing radiations, portability, and low cost [1,2]. One of the notable applications of MWI is towards breast cancer detection [3,4,5], with four clinical systems being tested in clinical trials [5]. The detection of breast cancer relies on the inherent dielectric contrast between normal and malignant breast tissues [6,7,8,9]. Besides breast cancer detection, various studies have employed MWI for the diagnosis of brain stroke, exploiting the dielectric contrast between ischemic and healthy tissues [10,11]. Recent studies have investigated the feasibility of using MWI for osteoporosis monitoring [2,12] based on the notable dielectric contrast between healthy and diseased human trabecular bones [13]. The dual-energy X-ray absorptiometry (DXA) is widely employed in clinical practices for bone health monitoring. However, DXA poses long-term health risks to the patients as it uses ionizing radiations up to 0.86 mrem [14]. Similarly, three-dimensional quantitative computed tomography (QCT) and high-resolution peripheral quantitative computed tomography (HR-pQCT) are rarely used in clinical practices due to the high-intensity ionizing radiations, expensive equipment, and cost of the test [15]. These imaging modalities provide high-resolution images and are clinically accepted. Contrary to this, MWI provides low-resolution images with the advantages of non-ionizing radiations, portability, and low cost, these clinical advantages and the dielectric contrast between healthy and diseased human trabecular bones make MWI a potential imaging modality for monitoring bone health in comparison to the DXA, QCT, and HR-pQCT [14,16].

MWI can be classified into two main categories: radar-based and tomographic MWI [17]. In radar-based MWI techniques, images are constructed based on the scattered waves that arise due to the dielectric contrast between normal and malignant tissues [6]. The radar-based techniques are mainly used to localize any strong scatterer/pathology in the biological tissues without reconstructing the full image of the biological tissues [18]. Contrary to this, the tomographic MWI techniques aim at retrieving the spatial distribution of dielectric properties (relative permittivity ($\varepsilon_{r}$) and conductivity ($\sigma \;\left(S/m\right)$)) of biological tissues by processing the measured scattered electromagnetic (EM) field data [19]. The tomographic-based MWI techniques are computationally expensive compared to the radar-based MWI techniques [19]. However, with the development of fast parallel tomography solutions, the computational cost of microwave tomography (MWT) approaches have reduced significantly [20].

The EM inverse scattering problem is inherently ill-posed and non-linear [1]. The regularization and linearization techniques are applied to deal with non-linearity and ill-posedness of the EM inverse scattering problem [19,21]. To this end, various non-linear iterative techniques have been proposed in the literature, such as the forward-backward time-stepping method [22], Gauss–Newton optimization approach [12,23], and microwave tomography using the dielectric Debye model [24]. The computational cost of these algorithms primarily depends upon the forward solver and the regularization techniques for the stabilization of the inversion method [19]. Moreover, the Gauss–Newton approaches are sensitive to the “initial guess”, which makes this approach less favorable in scenarios where less a priori information is available [25]. In EM inverse scattering problems, an “initial guess” provides the starting point of the convex optimization problem; hence, an inaccurate “initial guess” would lead to a solution that has no significance to the solution of the problem [1]. Besides non-linear iterative techniques, few linear approximation methods also exist, such as Born and Rytov approximations. These linear approximation methods help in reconstructing the dielectric properties of the targets that have lower dielectric contrast and small size [19]. In bone imaging applications, the dielectric contrast between cortical bone and trabecular bone is less [26]; therefore, the amount of energy penetrating trabecular bone is considerably higher than the reflected energy. Therefore, the contribution of measured scattered EM signals due to the trabecular bone would dominate the behavior of the objective function in the minimization problem.

The distorted Born iterative method (DBIM) is a well-known linear approximation technique for solving the EM inverse scattering problem [27,28]. The DBIM is an extension of Born and Rytov approximation and reconstructs the two-dimensional (2-D) and relatively high contrast imaging domain. The Born and Rytov approximations break down when the contrast of the imaging domain is relatively high, such as in the case of biological tissues [29]. In the Born approximation technique, the Green’s function is not updated at each iteration. However, in the DBIM approach, the Green’s function is updated at each iteration that makes it robust towards high contrast non-linear reconstruction problems [29]. The DBIM uses a succession of linear approximations to estimate the spatial distribution of dielectric properties of the reconstruction domain [30]. In this study, the underdetermined set of linear equations are solved by using an iterative method with adaptive thresholding for compressed sensing (IMATCS) during each DBIM iteration [31]. The IMATCS approach belongs from the family of compressed sensing (CS) methods, where the sparse signal is recovered from a lower-dimensional measurement vector, i.e., the number of measurements is much less than the number of signal entries [31]. Hence, CS techniques are suitable for determining the solution of an underdetermined system of linear equations based on the measurement matrix at each DBIM iteration [31].

Various other thresholding techniques exist for solving an underdetermined set of linear equations, such as iterative hard thresholding (IHT) [32], *K*-sparse algorithms, and iterative shrinkage thresholding algorithm (ISTA) [33]. The performance of these techniques primarily depends upon the threshold value and sparsity number as a priori information for signal recovery [31]. Similarly, the ISTA approaches are computationally expensive as they involve the selection of coefficients that maximize the correlation between propagation and scattering matrix at each iteration [30]. The IMATCS approach addresses the limitations of these techniques by picking the most significant signal entries at each iteration. In the IMATCS approach, a crude reconstruction is successively applied to the linear measurements of the signal. The recovered signal is then sparsed by employing an adaptive thresholding function [31]. The non-linearity and ill-posedness of the EM inverse scattering problem may cause unstable reconstructions of the target domain. Regularization techniques are applied to increase the robustness and to overcome the non-linearity of the EM inverse scattering problem [30]. Since the EM inverse scattering problem is approximated as linear in medical imaging applications; therefore, the IMATCS algorithm may diverge after some iterations. To address this limitation, an *L_2_*-regularization strategy is employed that leads to stable signal recovery. The *L_2_*-IMATCS performs better in scenarios where the IMATCS algorithm becomes unstable [31].

A comprehensive review of bone dielectric properties in the microwave frequency range by Amin et al. [2] reported that very few studies have measured dielectric properties of the human trabecular bones. Meaney et al. [12] reported in vivo dielectric properties of human calcaneus bone by using MWT for a frequency range of 900–1700 MHz for two patients suffering from lower leg injury [12]. Due to the limited sample size, no definite conclusion regarding the dielectric properties of human bones can be drawn from these results. Similarly, Meaney et al. [34] reported in vitro dielectric properties of porcine bone samples by using MWT for a frequency of 1100 MHz. In this study, the optimal results for the reconstruction of bone phantoms were found at 1 GHz. This frequency provided a good compromise between penetration depth and imaging resolution [35,36]. Furthermore, considering frequencies above 3 GHz for microwave bone imaging application would not be feasible due to the low penetration of EM waves [10]. Moreover, the fact that only a few studies have been conducted on the MWT of human bone motivates further studies on the characterization of human bone dielectric properties by using MWT. Moreover, no study has ever reported MWT of diseased human bone samples, which is of paramount importance for the development of EM-based diagnostic and therapeutic medical devices for bone diseases.

Two previous studies have performed the reconstruction of bone dielectric properties and both of these studies have reconstructed bone as a homogeneous tissue [12,37]. However, bone has a cortical and trabecular layer with significantly different dielectric properties [38]. No previous study has reported the reconstruction of the cortical and trabecular layer of the bone. This study aimed to assess whether the dielectric contrast between the cortical bone and trabecular bone is maintained in a simplistic imaging scenario and whether diseased trabecular bones can be differentiated based on the reconstruction of dielectric properties using MWT. Therefore, this work has considered a simplistic scenario of reconstructing a two-layered bone structure, where the outer layer mimics the dielectric properties of cortical bone and the inner layer mimics the dielectric properties of trabecular bone. This paper presents the implementation of the DBIM algorithm for the reconstruction of dielectrically accurate numerical bone phantoms. A total of seven bone phantoms were developed based on the single-pole Debye parameters of cortical bone and trabecular bone. A two-stage genetic algorithm (GA) [39] was used to fit the single-pole Debye model to the dielectric data of the cortical bone and trabecular bone obtained from Gabriel et al. [40]. Gabriel et al. [40] examined the (in vitro) dielectric properties of cortical bone and trabecular bone samples from porcine. This study has developed osteoporotic and osteoarthritis bone phantoms based on single-pole Debye parameters obtained from the dielectric measurements reported by Amin et al. [13]. The trabecular bone microarchitecture of osteoarthritis patients is compact and dense compared to osteoporotic patients [41]. The dense trabecular microarchitecture of bone indicates a higher degree of mineralization due to an increased amount of bone present [42,43]. Therefore, the bone samples from these two sets of patients allow the establishment of the variation in bone dielectric properties due to variation in the mineralization content and microarchitecture between two diseased bones. The *L_2_*-IMATCS approach reported by Azghani et al. [31] is employed as a linear solver in each DBIM iteration. The *L_2_*-IMATCS approach for reconstruction has shown promising results for a diverse range of dielectrically informed numerical bone phantoms. The initial findings on numerical tissue-mimicking phantoms have demonstrated that the osteoporotic and osteoarthritis human trabecular bones can be differentiated based on the spatial distribution of their reconstructed dielectric properties.

The remainder of the paper is organized as follows: Section 2 discusses the mathematical formulation of the adopted approach, including the DBIM formulation, IMATCS algorithm, the parameter selection in the IMATCS algorithm, and the design of numerical bone phantoms. Section 3 presents the results and discussion on the simulation testbed, the performance metrics to evaluate the reconstructed MWT images, the choice of IMATCS algorithm parameters, and the reconstruction of numerical bone phantoms. Finally, conclusions are drawn in Section 4.

## 2. Mathematical Formulation

The multiple scattering interactions in the heterogeneous target region cause non-linearity and the fact that the number of measurements is too small compared to the number of unknowns in the microwave inverse problem results in an ill-posed EM inverse scattering problem [44]. Therefore, non-linear optimization methods are used to estimate the dielectric properties of the target region from the measured EM scattered fields [44]. This involves an EM simulation along with the inversion of linear approximation of the EM field during each iteration of the algorithm. The unknown dielectric properties are estimated by using a parametric model of complex permittivity over the desired frequency band. To this end, this study implemented the DBIM approximation method, which linearizes the EM scattering wave equation by replacing the total field with a known incident field [21]. The incident field is estimated at each iteration of the DBIM algorithm in the presence of known background.

### 2.1. DBIM Formulation

**Figure 1:**
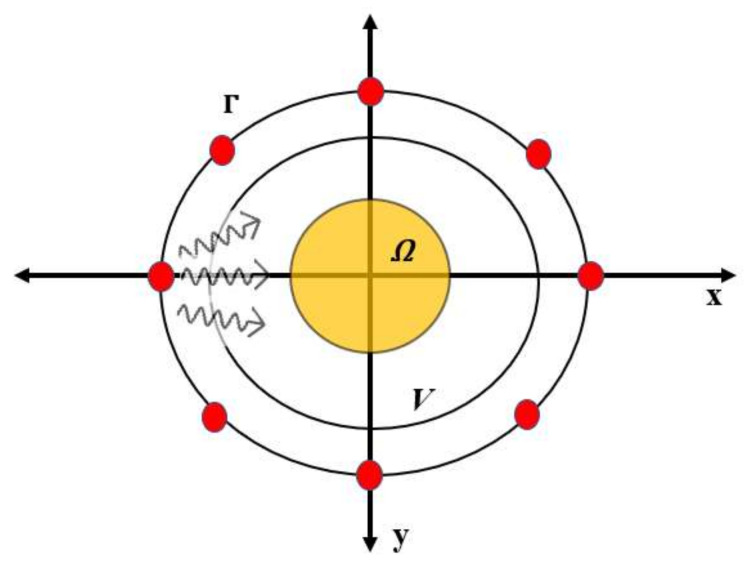
Microwave imaging scenario. Г denotes the contour with all EM sources, *V* denotes the overall imaging region, and *Ω* denotes the target to be imaged.

In an EM inverse scattering problem, a set of EM scattered fields are obtained from an unknown target region *Ω* as shown in Figure 1. A known EM source illuminates the imaging region *V* with the EM field. The resulting scattered field from the target region *Ω* is measured at one or more observation points outside the imaging region *V* as shown in Figure 1. The unknown complex permittivity ε in the imaging region *V* is estimated based on the measured scattered field and the complex permittivity of the background region. The integral equation of EM field at measurement point r and frequency ω can be expressed as:

(1) $$\Delta E_{s}\left(r,\omega \right)=\;E_{t}\left(r,\omega \right)\;–\;E_{i}\left(r,\omega \right),$$

(2) $$=\;\omega^{2}\mu \;\underset{V}{\int }G_{b\;}\left(r,\;r^{\prime },\omega \right)\delta \;\left(r^{\prime },\omega \right)E_{t}\left(r^{\prime },\omega \right)dr^{\prime },$$

where $\Delta E_{s}$ is the EM scattered field, $E_{t}$ is the total field, $E_{i}$ is the incident field in the presence of known background, $G_{b\;}$is the dyadic Green’s function for the background, and δ is the contrast function between the complex permittivity of the unknown region $\left(\varepsilon \left(r,\omega \right)\right)\;$and the dielectric profile of background $\left(\varepsilon_{b}\left(r,\omega \right)\right)$. Thus, Equation (2) represents the set of field measurements of the target region. The unknown of the objective function in Equation (2) is the contrast function $\left(\delta \left(r^{\prime },\omega \right)=\;\varepsilon \left(r^{\prime },\omega \right)-\;\varepsilon_{b}\left(r^{\prime },\omega \right)\right)$. The number of unknowns in the imaging region *V* are often greater than the number of measurements, which results in an undetermined system having no unique solution. Similarly, the Green’s function may not be available analytically when the background is not homogenous space [44]. Moreover, the total field within the imaging region *V* is unknown, which is a function of the complex permittivity of the unknown region, thus making the system non-linear in the unknown contrast function.

The DBIM method performs a succession of linear approximations to tackle the non-linearity of the EM inverse scattering problem given in Equation (2). At each DBIM iteration, the total field within the target region is approximated by the incident field, i.e., $E_{t}$ is replaced by $E_{i}$ in Equation (2). Each DBIM iteration involves the computation of the background field and Green’s function at the antennas and inside the imaging region *V*, which can be numerically computed by a *forward solution* of the EM scattering equation. During each DBIM iteration, the system of EM scattered equations obtained from the *forward solution* is inverted to obtain an approximate solution of the contrast between the target region and the current estimate of the background profile referred to as an *inverse solution*. The DBIM algorithm updates the background dielectric profile by iterating between *forward* and *inverse solutions* until convergence is reached in the minimization of residual scattering [44].

### 2.2. IMATCS Algorithm

In CS methods, the successful recovery of the signal primarily depends upon the measurement matrix. Various matrix transformation approaches are adopted, such as Toeplitz, Gaussian, and Bernoulli matrices, to transform the measurement matrix [31]. However, in the EM inverse scattering problem, the manipulation of the measurement matrix is not straightforward [31]. Moreover, the unknown complex permittivity vector is not sparse at each DBIM iteration. Finding a transformation matrix to induce sparsity in a complex permittivity vector is a challenging task. Furthermore, the EM equations are inherently non-linear and are approximated as linear as shown in Equation (2). To find the *inverse solution* in each DBIM iteration, this study employed the IMATCS method [31]. The IMATCS method belongs to the family of thresholding techniques. As discussed earlier, the threshold techniques need to be finely tuned based on a priori information of the underlying signal. However, many medical applications lack a priori information of the unknown signal. The IMATCS method addresses this limitation by using an adaptive threshold approach. The threshold value is exponentially decreased at each iteration of the IMATCS algorithm. The linear approximation of the EM integral equation given in Equation (1) can be expressed as:

(3) y=Mx,

where y represents the residual measurement data vector, M represents the measurement matrix having dimensions m × n (m < n), and x represents the unknown contrast function $\left(\delta \left(r^{\prime },\omega \right)=\;\varepsilon \left(r^{\prime },\omega \right)-\;\varepsilon_{b}\left(r^{\prime },\omega \right)\right)$ in terms of single-pole Debye parameters. The measurement matrix M is constructed by the incident field and the dyadic Green’s function for the background and is updated at each DBIM iteration. The aim in Equation (3) is to find x from y, subject to the condition that the number of measurements m are less than the number of unknowns n. The considered problem expressed in Equation (3) can be solved by using the IMATCS method and can be expressed as:

(4) $$\underset{x}{\min}∥y-Mx{∥}_{2}^{2}\;+\;\beta ∥x{∥}_{0}.$$

The solution of Equation (4) for the adaptive IMATCS approach can be written as:

(5) $$x_{j+1}=A_{0}e^{-\alpha i}\left(x_{k}+\;\beta M^{*}\;\left(y-Mx_{k}\right)\right),$$

where $M^{*}$ is the conjugate transpose of M, β controls the convergence of the algorithm, $A_{0}$ is the threshold value, $x_{k}$ is the unknown coefficient vector, α is the threshold step size, and j is the iteration number. The algorithm given in Equation (5) starts with a null initial value, i.e., $x_{0}=0$. The $x_{j}$ is recovered after the specified number of IMATCS iterations. The adaptive threshold enables the recovery of $x_{j}$ from the linear measurements without any a priori information of the signal [31].

The IMATCS method finds $x_{j}$ from the set of measurements shown in Equation (2). However, these measurements are not linear and are approximated as linear in MWI applications. Therefore, this assumption may lead to instability and divergence of some IMATCS iterations. To address this limitation, an $L_{2}$-regularized $L_{0}$-minimization approach is adopted as reported by Azghani et al. [31]. The optimization problem given in Equation (4) can be re-casted as:

(6) $$\underset{x}{\min}∥y-Mx{∥}_{2}^{2}\;+\;\beta_{1}∥x{∥}_{0}\;+\;\beta_{2}∥x{∥}_{2}^{2}\;.$$

The solution of Equation (6) for the adaptive IMATCS approach can be written as:

(7) $$x_{j+1}=\frac{1}{1+\;\beta_{2}\;}A_{0}e^{-\alpha i}\left(x_{k}+\;\beta_{1}M^{*}\;\left(y-Mx_{k}\right)\right).$$

The instability caused in the IMATCS approach due to the linear assumption of measurements in Equation (2) is addressed by the $L_{0}/L_{2}$, minimization/regularization approach. The $L_{2}$-regularization approach is derived from the $L_{2}$-IHT method. The $L_{2}$-IHT method is extremely sensitive towards the proper selection of the threshold value and therefore does not result in convergence of an acceptable solution [31]. However, the $L_{2}$-IMATCS approach provides a stable and better recovery of $x_{j}$ from the linear measurements given in Equation (2). The $L_{2}$-IMATCS approach for the *inverse solution* in each DBIM iteration is given in Algorithm 1.
**Algorithm 1**$L_{2}$-IMATCS**1:** The measurement matrix M updates at each DBIM iteration **2:**
**Input**: The measurement matrix M  The residual measurement data vector y **3:**
**Output**: The contrast function δ **4:**
**Method:**
$L_{2}$-IMATCS       $x_{0}\leftarrow 0$        **for**
*j =* 1: *itrmax*         *Thresh*
←
$A_{0}e^{-\alpha j}$    $x_{j}\leftarrow \frac{1}{1+\;\beta_{2}\;}Thresh\left(x_{j-1}+\;\beta_{1}M^{*}\;\left(y-Mx_{j-1}\right)\right)$        **end for**  **End method** **5:** Update the contrast function δ based on $x_{j}$ **6:** Return to **1**

### 2.3. Parameter Selection of IMATCS Algorithm

The optimal solution of $x_{j}$ from the $L_{2}$-IMATCS approach primarily depends upon the choice of regularization parameters $\beta_{1}$ and $\beta_{2}$, the threshold value $A_{0}$, the threshold step size α, and the maximum iterations of the IMATCS algorithm [31]. The parameter $\beta_{1}$ controls the convergence of the algorithm and is subject to the following condition:

(8) $$0\;\leq \;\beta_{1}\;\leq \;\frac{2}{\gamma_{max}\left(MM^{*}\right)}\;,$$

where $\gamma_{max}\left(MM^{*}\right)$ is the maximum value of eigenvalues of the product of M and its conjugate. The measurement matrix M updates in each DBIM iteration. For the reconstruction of dielectric properties of bone in the following section, the value of $\beta_{1}$ is set as:

(9) $$\;\beta_{1}\;=\;\frac{1.9}{\gamma_{max}\left(MM^{*}\right)}\;.$$

The choice of the threshold value and maximum iterations of the IMATCS algorithm influence the quality and resolution of the reconstructed image. The selection of the threshold value and maximum iterations of the IMATCS algorithm was performed empirically for the case of reconstruction of bone dielectric properties. The results are reported in the following section. Similarly, the value of the threshold step size was set to equal 0.01 for the reconstruction of all considered bone phantoms. The value of the threshold step size was found empirically. The small step size of the threshold step size allows a slow decrease in the threshold value for each IMATCS iteration, thus capturing almost all significant components of the signal.

### 2.4. Numerical Bone Phantoms

A two-layered circular model of bone was developed. The outer layer represents the cortical bone and the inner layer represents the trabecular bone. This study considered a total of seven bone phantoms. The outer layer in all phantoms represents the cortical bone. The inner layer that represents the trabecular bone was varied to mimic the natural variation of dielectric properties reported in the literature for various clinical conditions. The sequence of bone tissues for outer and inner layers with their corresponding labels are tabulated in Table 1.

The two-layered circular model was transformed into an electromagnetic model based on the single-pole Debye parameters of each layer. The frequency dependence of complex permittivity of biological tissues can be modeled by using a single-pole Debye model over the frequency range of interest (0.5–8.5 GHz). The single-pole Debye model can be expressed as:

(10) $$\varepsilon_{r}\;\left(\omega \right)=\;\varepsilon_{\infty }+\;\frac{\Delta \varepsilon }{1+j\omega Ʈ}\;+\;\frac{\sigma_{s}}{j\omega \varepsilon_{0}},$$

where $\varepsilon_{\infty }$ is the permittivity value at the highest frequency under consideration, Δε is the difference between the permittivity value at the highest and lowest frequency values under consideration, $\sigma_{s}$ is the conductivity, and Ʈ is the relaxation time constant. The parametric values of the Debye parameters for the considered bone tissues are tabulated in Table 2 for the frequency range of 0.5–8.5 GHz. Moreover, the values of relative permittivity and conductivity are listed in Table 2 for the frequency of 1 GHz. To simplify the finite difference time domain (FDTD) simulation, the relaxation time constant was considered spatially invariant with a constant value of 0.5 ps. A two-stage genetic algorithm (GA) was used to fit the single-pole Debye model to the measured data obtained from Gabriel et al. [40] for bone phantom P1. Moreover, the single-pole Debye parameters were determined for osteoporotic and osteoarthritis human trabecular bones based on the dielectric properties reported by Amin et al. [13]. Lazebnik et al. [45] proposed single-pole Debye parameters for breast tissues at the microwave frequency range. These parameters are widely employed for microwave breast imaging. However, no study to date has ever proposed single-pole Debye parameters for cortical bone and trabecular bone. To this end, this study determined single-pole Debye parameters for the evaluation of bone phantoms.

## 3. Results and Discussion

This section presents the results obtained by the $L_{2}$-IMATCS approach for bone dielectric properties’ reconstruction using dielectrically informed numerical models.

### 3.1. Simulation Testbed

**Figure 2:**
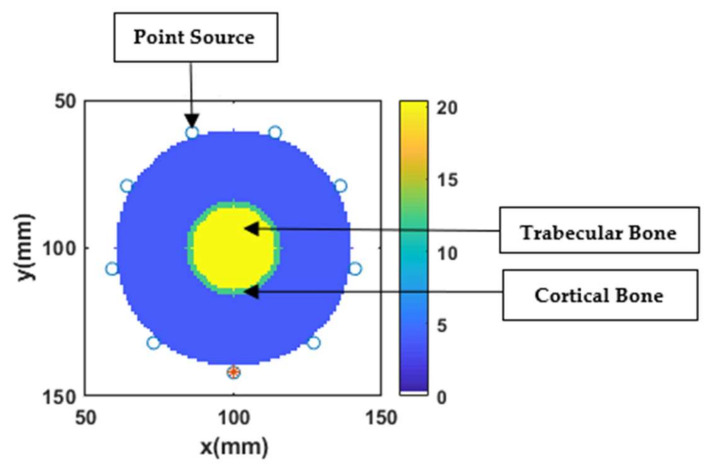
Simulation testbed.

The measured data were collected by simulating the model of the imaging system using the FDTD method with a uniform grid cell size of 1 mm. The FDTD simulation was also used as a forward solver for the inversion process as commonly used in previous studies that investigated the breast numerical phantoms [1,19,30,31,44]. In all our simulation testbeds, the bone phantoms were assumed to be immersed in a lossless non-dispersive matching medium whose single-pole Debye parameters are $\varepsilon_{\infty }=\;2.848,\;\Delta \varepsilon =\;1.104,\;\sigma_{s}=\;0.005\;S/m$. The evaluated bone phantoms, simulations, and reconstructions are performed for 2-D imaging scenarios. To gain maximum coverage and optimal resolution, a total of nine ideal dipole antennas were placed in a circular array across the bone phantoms as shown in Figure 2. For the 2-D geometry, the Hertzian dipole antennas correspond to point sources. These point sources were equally spaced around the bone phantom in a circular array of the radius of 12 cm. These point sources sequentially illuminated the bone phantoms with a modulated wideband Gaussian pulse with a center frequency of 1 GHz and −3-dB bandwidth. The scattered EM signals from bone phantoms were recorded, and unique measurements for each transmit-receive antenna pair were recorded. All the redundant data from reciprocal channels and monostatic channels were not recorded. The $L_{2}$-IMATCS approach reconstructs the single-pole Debye parameters, which were then converted into a complex permittivity profile of bone. To avoid “inverse crime”, the simulated data were corrupted with additive Gaussian noise (AWGN) [1].

### 3.2. Performance Metrics

Two scalar metrics are considered in this study to perform a quantitative comparison between the reconstructed and corresponding reference bone dielectric properties. The first scalar metric measures the error based on the normalized root mean square error (NRMSE) between the complex permittivity of reference bone phantom and reconstructed bone phantom. The NRMSE is defined as:

(11) NRMSE= ∥εr f − ε^rf∥L22 ∥εr f∥L22,

where $\varepsilon_{r}\;\left(f\right)$ is the complex permittivity profile of reference bone dielectric properties, and ${\hat{\varepsilon }}_{r}\left(f\right)$ is the complex permittivity profile of reconstructed bone dielectric properties. The NRMSE is separately calculated for both the real $(\varepsilon^{\prime }$) and imaginary ($\varepsilon^{\prime \prime }$) parts of the complex permittivity profile for all bone phantoms. The results of NRMSE for the real and imaginary parts of complex permittivity for all reconstructed bone phantoms are tabulated in Table 3. For a fair comparison, the NRMSE is calculated for the two-layered circular bone phantom inside the imaging domain *V*. The application of the regularization approach resulted in smooth reconstructed complex permittivity profiles, thus resulting in lower error values between the reference and reconstructed bone phantoms. Similar values of NRMSE were reported by Ambrosanio et al. [30] for the reconstruction of 2-D numerical heterogeneous breast phantoms. The authors proposed an adaptive multi-threshold ISTA (AMTISTA) approach for the reconstruction of breast phantoms.

The second performance metric, the structural similarity index (SSIM), intends to correlate the structural similarity between the reconstructed and reference bone dielectric properties [46]. To this end, SSIM compares two images at a time. The SSIM considers the luminance, contrast, and structure to produce a similarity value between the two images [6]. The SSIM values range between 0 and 1, a value of 0 indicates that no structural similarity exists between the two images under comparison; however, a value of 1 indicates maximum similarity between the two images under comparison. The results of SSIM between the real and imaginary parts of the reference and reconstructed complex permittivity profiles are tabulated in Table 4. Based on the SSIM values in Table 4, it can be observed that the reference and reconstructed bone dielectric properties have high similarity in terms of the real and imaginary parts of complex permittivity for each considered bone phantom.

### 3.3. Choice of Number of IMATCS iterations, Number of DBIM iterations, and Threshold ($A_{0}$)

**Figure 3:**
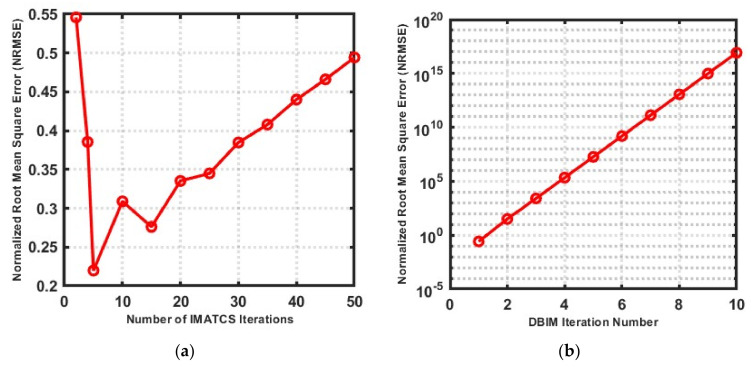

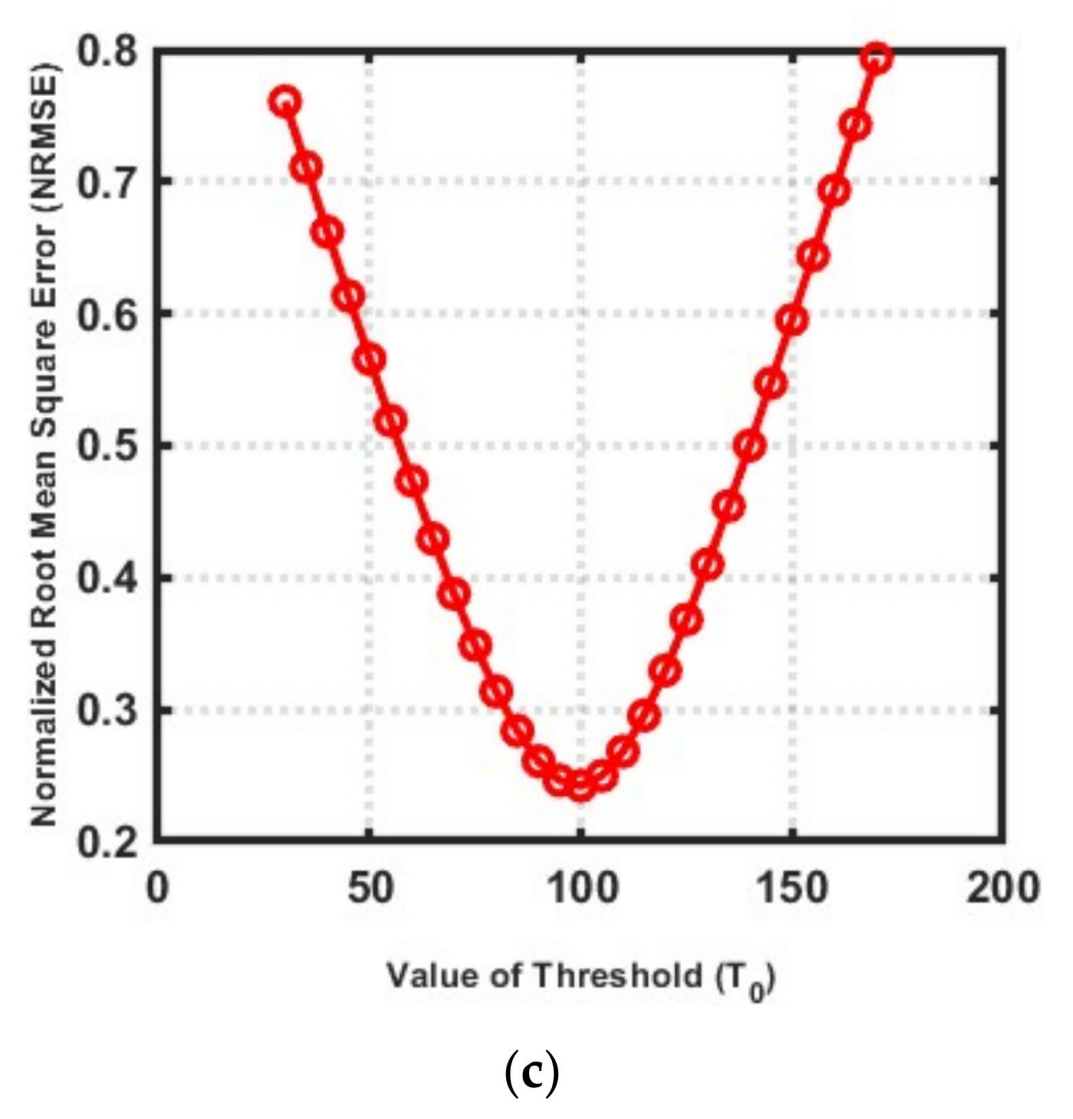
Relationship between NRMSE and (**a**) number of IMATCS iterations, (**b**) number of DBIM iterations, and (**c**) value of threshold ($A_{0}$).

The choice of the number of IMATCS iterations, number of DBIM iterations, and threshold ($A_{0}$) has a significant impact on the resolution and quality of the reconstructed complex permittivity profile of bone. For the optimal selection of these parameters, a numerical analysis was performed on bone phantom P1. The complex permittivity profile of bone phantom P1 was reconstructed for the different number of IMATCS iterations while keeping a fixed number of DBIM iterations. The NRMSE values were calculated for the real part of the complex permittivity of reconstructed bone phantom P1 for each simulation as shown in Figure 3a. The minimum value of NRMSE was obtained for five IMATCS iterations. It can be observed from Figure 3a that the NRMSE increases as the number of IMATCS iterations increase; similarly, a higher trend of NRMSE values was observed for IMATCS iterations less than five. Therefore, for the reconstruction of all bone phantoms, five IMATCS iterations were used. Similarly, for the optimal number of DBIM iterations, the bone phantom 1 was simulated for the different number of DBIM iterations for a fixed number of IMATCS iterations (five). The NRMSE values were calculated for the real part of the complex permittivity of the reconstructed bone phantom P1 as shown in Figure 3b. It can be observed from Figure 3b that the NRMSE increases as the number of DBIM iterations increases. This is because no constraint on the upper limit of values was incorporated on the Debye parameters after each DBIM iteration, which results in saturation of the estimated Debye parameters. Moreover, the value of the threshold remains fixed in the IMATCS approach for solving an underdetermined set of linear equations, which results in the saturation of most of the components of the updating contrast function. Therefore, the value of NRMSE increases significantly after the first DBIM iteration. The minimum value of NRMSE was found for the first DBIM iteration. Therefore, the reconstruction of all bone phantoms in this study was obtained for the first DBIM iteration.

To find the optimal value for the threshold value $A_{0}$, the bone phantom P1 was simulated for different values of the threshold for a fixed number of IMATCS (five) and DBIM iterations (one). The NRMSE values were calculated for the real part of the reconstructed complex permittivity profile as shown in Figure 3c. Based on the NRMSE values, it can be observed from Figure 3c that the minimum values of NRMSE resulted for a threshold in the range of 90–100. Moreover, it can be observed that the values of NRMSE increase for a threshold greater than 100. Similarly, for thresholds less than 90, the values of NRMSE are found on the higher side. Therefore, for the reconstruction of all considered bone phantoms in this study, the value of the threshold was kept in the range of 90–100.

### 3.4. Reconstruction of Numerical Bone Phantom 1 (P1)

**Figure 4:**
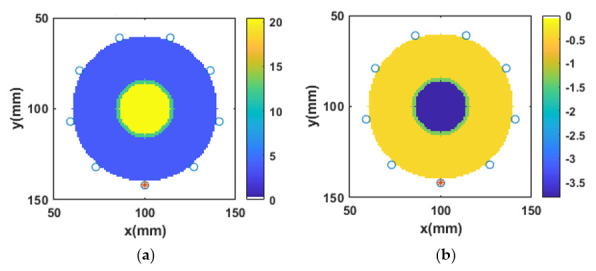

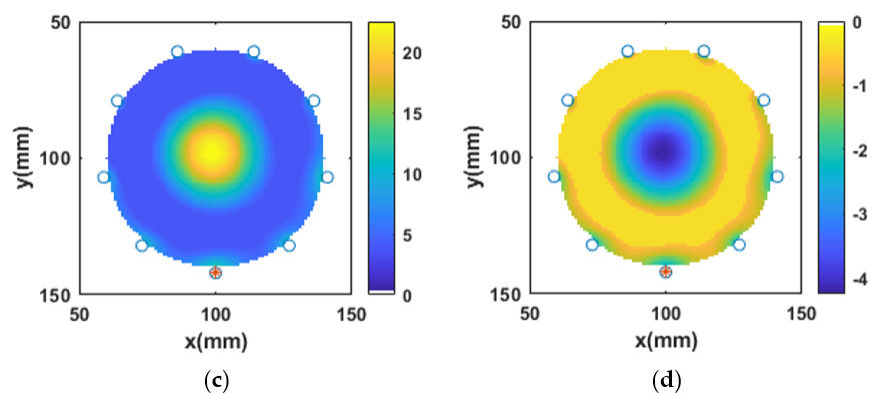
Real and imaginary parts of complex permittivity of (**a**) and (**b**) reference P1, (**c**) and (**d**) reconstructed P1 at 1 GHz.

As discussed, a two-layered circular model of bone was developed as shown in Figure 4a,b, representing the reference real and imaginary parts of the complex permittivity of bone phantom P1, respectively. The outer green layer is assigned the dielectric properties of the cortical bone and the inner yellow layer is assigned the dielectric properties of the trabecular bone. The point sources were directly in contact with the imaging region *V*. For all bone phantoms, initially, the single-pole Debye parameters were reconstructed at 1 GHz. The reconstructed Debye parameters were then transformed into the complex permittivity profile. The value of $\beta_{2}$ was set to 0.005 for the reconstruction of bone dielectric properties. No a priori information was used for the $L_{2}$-IMATCS approach for the reconstruction of bone dielectric properties. The reconstructed real and imaginary parts of the complex permittivity of bone phantom P1 are shown in Figure 4c,d respectively. Comparing the reference and reconstructed real and imaginary parts of complex permittivity, it can be observed that the shape and size of bone phantom P1 remain intact after the reconstruction. Moreover, the reconstructed real and imaginary part of the complex permittivity of bone phantom P1 suggests that good reconstructions of bone dielectric properties can be achieved by using the $L_{2}$-IMATCS approach, resulting in lower values of NRMSE for the real and imaginary parts of the complex permittivity as tabulated in Table 3.

### 3.5. Reconstruction of Numerical Bone Phantom 2, 3, and 4 (P2, P3, P4)

**Figure 5:**
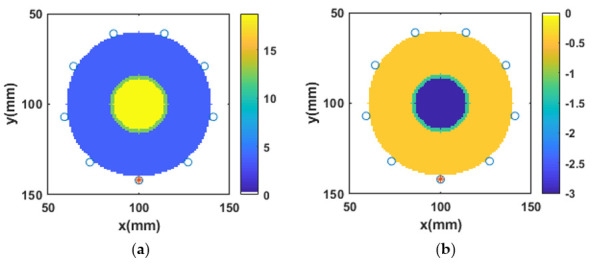

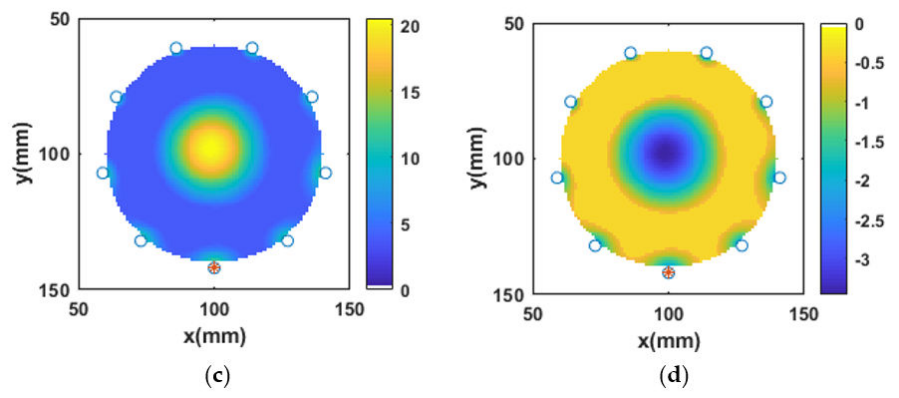
Real and imaginary parts of complex permittivity of (**a**) and (**b**) reference P2, (**c**) and (**d**) reconstructed P2 at 1 GHz.

The bone phantoms P2, P3, and P4 were categorized as osteoporotic bone phantoms. Like P1; P2, P3, and P4 were designed as two-layered circular bone models. The outer layer was assigned the dielectric properties of the cortical bone, whereas the dielectric properties of the inner trabecular bone layer were varied to account for the natural variation of dielectric properties of osteoporotic bones reported by Amin et al. [13]. The bone phantom P2 was designed to mimic the mean dielectric properties of osteoporotic bones, bone phantom P3 was assigned the lower-bound dielectric properties of osteoporotic bones, and bone phantom P4 was assigned the upper-bound dielectric properties of osteoporotic bones. The lower- and upper-bound dielectric properties of osteoporotic bones were considered to investigate the robustness of the $L_{2}$-IMATCS approach for the reconstruction of dielectric properties of diverse bone phantoms. Moreover, to investigate the fact that the reconstruction of complex permittivity profiles of bone phantoms P2, P3, and P4 do not overlap with each other, the reconstructed complex permittivity profiles kept the natural variation of dielectric properties of osteoporotic bones intact. The visual images for reconstructed complex permittivity profiles for bone phantoms P2, P3, and P4 are similar; therefore, only the reconstructed images of bone phantom P2 are shown here. Figure 5a,b, represents the reference real and imaginary part of the complex permittivity of bone phantom P2, respectively. The simulation setup was kept the same for the reconstruction of all bone phantoms as described for P1. The reconstructed real and imaginary parts of the complex permittivity of P2 are shown in Figure 5c,d, respectively. Comparing the reference and reconstructed real and imaginary parts of the complex permittivity of bone phantom P2, it can be observed that good reconstructions of bone dielectric properties are achieved by using the $L_{2}$-IMATCS approach. The values of NRMSE and SSIM for the bone phantoms P2, P3, and P4 were calculated for the real and imaginary parts of the complex permittivity and are tabulated in Table 3 and Table 4, respectively.

### 3.6. Reconstruction of Numerical Bone Phantom 5, 6, and 7 (P5, P6, P7)

**Figure 6:**
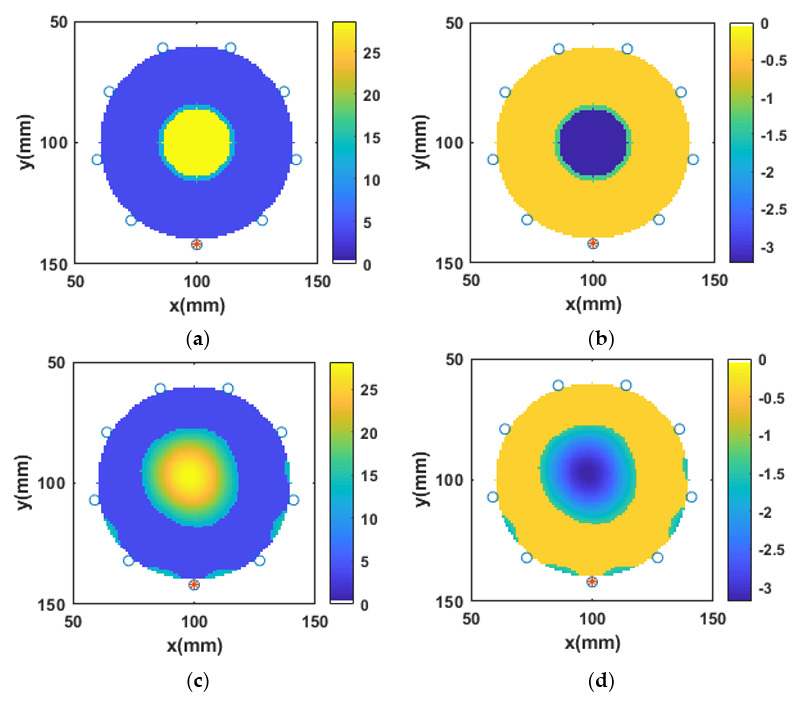
Real and imaginary parts of complex permittivity of (**a**) and (**b**) reference P5, (**c**) and (**d**) reconstructed P5 at 1 GHz.

The bone phantoms P5, P6, and P7 were categorized as osteoarthritis bone phantoms. Like P1, P5, P6, and P7 were designed as two-layered circular bone models. The outer layer was assigned the dielectric properties of the cortical bone, whereas the dielectric properties of the inner trabecular bone layer were varied to account for the natural variation of dielectric properties of osteoarthritis bones reported by Amin et al. [13]. The bone phantom P5 was designed to mimic the mean dielectric properties of osteoarthritis bones, bone phantom P6 was assigned the lower-bound dielectric properties of osteoarthritis bones, and bone phantom P7 was assigned the upper-bound dielectric properties of osteoarthritis bones. As for the case of osteoporotic bone phantoms, the $L_{2}$-IMATCS approach provides robustness for the reconstruction of the complex permittivity profile of bone phantoms P5, P6, and P7. Moreover, the reconstruction of complex permittivity profiles of bone phantoms P5, P6, and P7 do not overlap with each other and the reconstructed complex permittivity profiles keep the natural variation of dielectric properties of osteoarthritis bones intact. The mean values of single-pole Debye parameters for osteoarthritis bones, as tabulated in Table 2, are higher as compared to osteoporotic bones. The contrast between single-pole Debye parameters between outer and inner layers of bone phantoms P5, P6, and P7, is higher compared to the bone phantoms P2, P3, and P4. The visual images for reconstructed complex permittivity profiles for the bone phantoms P5, P6, and P7 are similar; therefore, only the reconstructed images of bone phantom P5 are shown here. Figure 6a,b, represents the reference real and imaginary parts of the complex permittivity of bone phantom P5, respectively. The reconstructed real and imaginary parts of the complex permittivity of bone phantom P5 are shown in Figure 6c,d, respectively. Comparing the reference and reconstructed real and imaginary parts of the complex permittivity parts of bone phantom P5, it can be observed that good reconstruction of numerical bone phantoms can be achieved by using the $L_{2}$-IMATCS approach, even for higher contrast two-layered bone phantoms. The values of NRMSE and SSIM for bone phantoms P5, P6, and P7 were calculated for the real and imaginary parts of the complex permittivity and are tabulated in Table 3 and Table 4, respectively.

### 3.7. Robustness of $L_{2}$-IMATCS for Reconstruction of Bone Phantoms P1, P2, P3, P4, P5, P6, and P7

**Figure 7:**
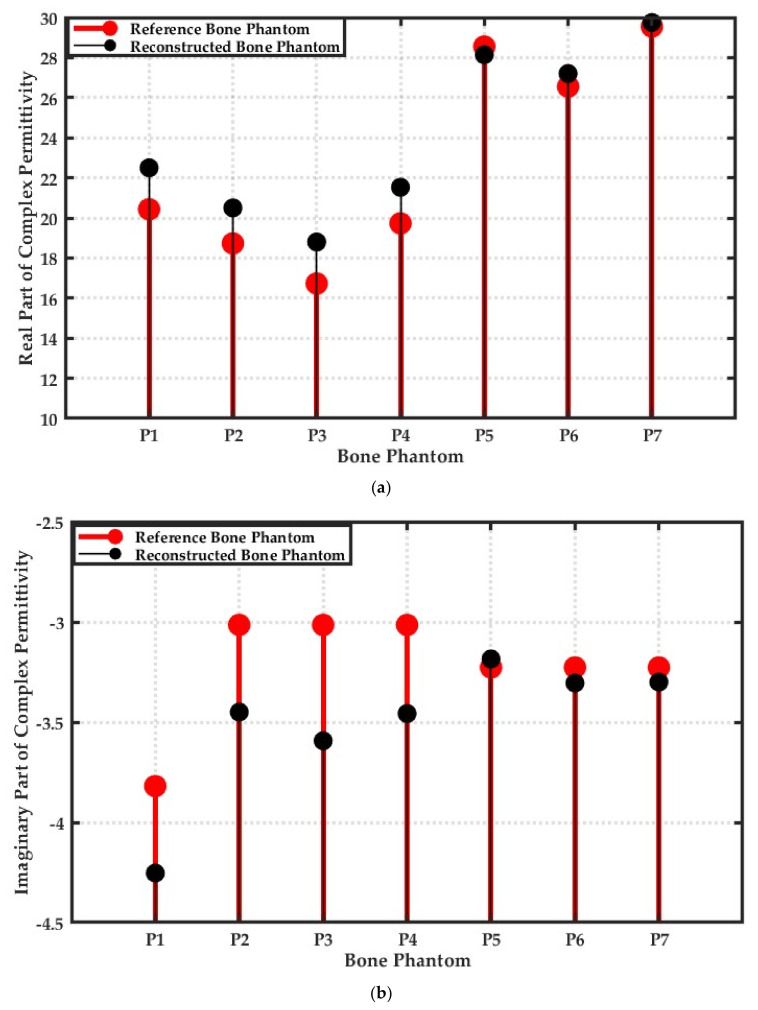
(**a**) Peak values of the real part of complex permittivity of reconstructed and reference bone phantoms (**b**) Peak values of the imaginary part of complex permittivity of reconstructed and reference bone phantoms at 1 GHz.

To investigate the robustness of the $L_{2}$-IMATCS approach for all considered bone phantoms, an analysis was performed to compare the peak values of the complex permittivity profile of reference and reconstructed bone phantoms. Figure 7a,b represents the comparison of the peak value of real and imaginary parts of the complex permittivity of all considered bone phantoms, respectively. The red-filled dots in the scatter plot represent the peak value of the complex permittivity of the reference bone phantom, whereas the black-filled dots represent the peak value of the reconstructed complex permittivity for each bone phantom. It can be observed from Figure 7a,b that the differentiation between the different diseased bones is possible using the real part of the reconstructed complex permittivity of bone phantoms. The reconstructed peak values of the complex permittivity of each bone phantom compared to the reference profile ensures the robustness of the $L_{2}$-IMATCS approach for the reconstruction of diverse bone phantoms. Hence, the adopted approach for the differentiation of osteoporotic and osteoarthritis bone phantoms can be employed for bone health monitoring. The next step involves investigating the robustness of the $L_{2}$-IMATCS approach for the experimental data, where the measurements will be performed on anatomically realistic bone phantoms.

### 3.8. Relative Error between Reference and Reconstructed Numerical Bone Phantoms

**Figure 8:**
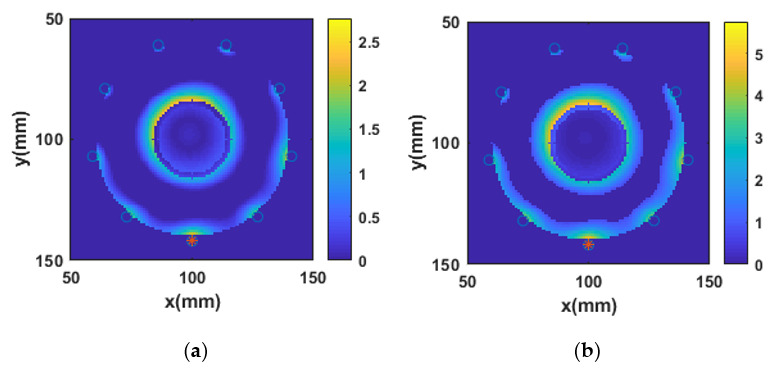
Relative error maps for (**a**) Real part of complex permittivity (**b**) Imaginary part of complex permittivity for bone phantom P1 at 1 GHz.

Figure 8a,b represents the relative error map for the real and imaginary parts of the complex permittivity of bone phantom P1, respectively. The reference and reconstructed real and imaginary parts of the complex permittivity of bone phantom P1 are shown in Figure 4. It can be observed from Figure 8a,b that the relative error between the reference and reconstructed real and imaginary parts of the complex permittivity is low in general. However, as expected, the error is larger at the edges of the bone phantom. There are two reasons for observing large relative error at the edges or boundary areas: the first reason is the expected large EM field perturbation at the boundaries of two mediums with different dielectric properties, and the second reason is due to a small localization error in the reconstructed dielectric profile. Moreover, the error is small for the real part of complex permittivity compared to the imaginary part of complex permittivity. The lower error values suggest that the reconstructed real and imaginary parts of the complex permittivity of bone phantom P1 can be achieved by using the $L_{2}$-IMATCS approach. A similar effect on the boundaries was found for all seven bone phantoms.

**Figure 9:**
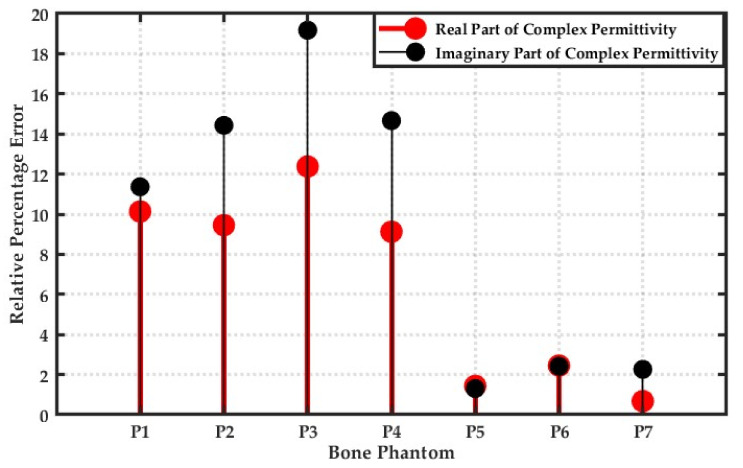
Relative percentage error between peak values of reference and reconstructed real and imaginary parts of complex permittivity at 1 GHz for all bone phantoms.

To investigate the relative error for all considered bone phantoms, an analysis was performed to calculate the relative error between peak values of the real and imaginary parts of the complex permittivity of the reference and reconstructed bone phantoms. Figure 9 represents the relative error between the peak values of the real and imaginary part of the complex permittivity of all considered bone phantoms. The red-filled dots represent the relative error between the peak values of the real part of complex permittivity, whereas the black-filled dots represent the relative error between the peak values of the imaginary part of the complex permittivity for each bone phantom. It can be observed from Figure 9 that the relative error is found to be less for the real part compared to the imaginary part of the complex permittivity.

### 3.9. Impact of Signal-to-Noise Ratio (SNR) on Reconstructed Numerical Bone Phantoms

To evaluate the robustness of the $L_{2}$-IMATCS approach in noisy scenarios, and to avoid “inverse crime”, the total field calculated from bone phantom simulations was corrupted with AWGN [1]. The variation of NRMSE on the reconstructed complex permittivity profile is observed for a range of SNR values. The values of SNR range from 20 to 60 dB with a step size of 10 dB. An individual experiment was performed for each considered bone phantom for a specific value of SNR. The total field calculated from each bone phantom was corrupted with the corresponding value of SNR relative to the energy of the total field. To this end, a total of 35 numerical experiments (7 numerical phantoms × 5 SNR levels) were performed. The procedure adopted for the assessment of SNR on reconstructed numerical bone phantoms is the same as that reported in the literature [1,19,30,31,44]. The NRMSE was calculated between the reference and reconstructed complex permittivity profiles for all bone phantoms in the presence of SNR. The results are tabulated in Table 5 and Table 6. It can be observed from Table 5 and Table 6, where even for low SNR values of 20 dB relative to the total received signal, the reconstruction errors are quite low both for the real and imaginary parts of the complex permittivity profile. The low reconstruction error values ensure the robustness of the $L_{2}$-IMATCS approach in the presence of AWGN.

## 4. Conclusions

The evaluation of MWI methods on numerical phantoms before clinical testing of the MWI system provides a controlled realistic imaging scenario. Moreover, it helps to evaluate the robustness of the adopted approach for the reconstruction of dielectric properties under realistic imaging scenarios. This study considered seven diverse numerical bone phantoms with accurate dielectric properties of different diseased human bones. To reconstruct the dielectric properties, a DBIM-based MWT approach was adopted in conjunction with the $L_{2}$-IMATCS approach.

The results of reconstructed bone dielectric properties showed that the adopted approach for linear inversion provides good reconstruction in comparison to the reference bone dielectric properties even for low SNR values. The results showed that the osteoporotic and osteoarthritis bones can be differentiated based on reconstructed complex permittivity profiles. The objective of this study was to evaluate MWT for the assessment of different diseased bone phantoms for bone health monitoring. The reported NRMSE between reference and reconstructed bone dielectric properties is in agreement with literature reporting the $L_{2}$-IMATCS approach for reconstruction of numerical breast phantoms.

This study reconstructed the dielectric properties of simplified 2-D bone phantoms. While the considered scenario is rather simplistic, it demonstrates the feasibility of reconstruction of bone dielectric properties using the proposed method. Future studies will extend the adopted approach to more realistic three-dimensional imaging scenarios in addition to the evaluation of the proposed methods on experimental data obtained from anatomically realistic bone phantoms measured with the MWI system. Further, a more proper decomposition basis will be investigated for enforcing sparsity to reduce the ill-posedness of the EM inverse scattering problem.

## Figures and Tables

**Table 1:** Numerical bone phantoms for simulations.

PL	OBTL	IBTL
P1	Cortical Bone	Trabecular Bone
P2	Cortical Bone	Osteoporotic Bone Mean
P3	Cortical Bone	Osteoporotic Bone Lower Bound
P4	Cortical Bone	Osteoporotic Bone Upper Bound
P5	Cortical Bone	Osteoarthritis Bone Mean
P6	Cortical Bone	Osteoarthritis Bone Lower Bound
P7	Cortical Bone	Osteoarthritis Bone Upper Bound

PL = Phantom Label, OBTL = Outer Bone Tissue Layer, IBTL = Inner Bone Tissue Layer.

**Table 2:** Single-pole Debye parameters of bone tissues. The values of $\varepsilon_{r}$ and σ are given for 1 GHz.

Tissue	$\varepsilon_{\infty }$	Δε	$\sigma_{s}\left(S/m\right)$	$\varepsilon_{r}$	$\sigma \left(S/m\right)$
Cortical Bone	8.75	4	0.01	12.39	0.0736
Trabecular Bone	14	7	0.1	20.43	0.2125
Osteoporotic Bone Mean	16	3	0.12	18.73	0.1677
Osteoporotic Bone Lower Bound	14	3	0.12	16.73	0.1677
Osteoporotic Bone Upper Bound	17	3	0.12	19.73	0.1677
Osteoarthritis Bone Mean	24	5	0.1	28.55	0.1795
Osteoarthritis Bone Lower Bound	22	5	0.1	26.55	0.1795
Osteoarthritis Bone Upper Bound	25	5	0.1	29.55	0.1795

**Table 3:** NRMSE between original and reconstructed bone phantoms.

Phantom	NRMSE	
	$\varepsilon^{\prime }$	$\varepsilon^{\prime \prime }$
P1	0.212	0.253
P2	0.239	0.228
P3	0.249	0.228
P4	0.226	0.222
P5	0.246	0.252
P6	0.228	0.242
P7	0.242	0.245

**Table 4:** SSIM between original and reconstructed bone phantoms.

Phantom	SSIM	
	$\varepsilon^{\prime }$	$\varepsilon^{\prime \prime }$
P1	0.973	0.995
P2	0.968	0.997
P3	0.959	0.997
P4	0.971	0.997
P5	0.959	0.993
P6	0.966	0.994
P7	0.953	0.992

**Table 5:** NRMSE between original and reconstructed bone phantoms for the real part of complex permittivity.

SNR (dB)	P1	P2	P3	P4	P5	P6	P7
20	0.224	0.239	0.249	0.228	0.247	0.228	0.244
30	0.220	0.238	0.249	0.226	0.245	0.229	0.243
40	0.220	0.239	0.249	0.226	0.245	0.229	0.242
50	0.220	0.239	0.249	0.226	0.247	0.228	0.243
60	0.220	0.239	0.249	0.226	0.246	0.228	0.243

**Table 6:** NRMSE between original and reconstructed bone phantoms for the imaginary part of complex permittivity.

SNR (dB)	P1	P2	P3	P4	P5	P6	P7
20	0.256	0.229	0.228	0.223	0.253	0.241	0.247
30	0.254	0.229	0.229	0.222	0.252	0.243	0.246
40	0.253	0.228	0.228	0.221	0.252	0.242	0.245
50	0.253	0.228	0.228	0.222	0.252	0.242	0.245
60	0.253	0.228	0.228	0.222	0.252	0.242	0.245
